# Potential Therapeutic Anti-Inflammatory and Immunomodulatory Effects of Dihydroflavones, Flavones, and Flavonols

**DOI:** 10.3390/molecules25041017

**Published:** 2020-02-24

**Authors:** Cristina Zaragozá, Lucinda Villaescusa, Jorge Monserrat, Francisco Zaragozá, Melchor Álvarez-Mon

**Affiliations:** 1 Biomedical Sciences Department, Pharmacology Unit, University of Alcalá, Alcalá de Henares, 28871 Madrid, Spain; lucinda.villaescusa@uah.es (L.V.); francisco.zaragoza@uah.es (F.Z.); 2 Laboratory of Immune System Diseases and Oncology, Department of Medicine and Medical Specialties, University of Alcalá, Alcala de Henares, 28871 Madrid, Spain; jorge.monserrat@uah.es (J.M.); mademons@gmail.com (M.Á.-M.); 3 Immune System Diseases and Oncology Service, University Hospital “Príncipe de Asturias”, Alcalá de Henares, 28871 Madrid, Spain; 4 Biomedical Research Networking Center in Hepatic and Digestive Diseases (CIBERehd), 28871 Madrid, Spain; 5 Ramón y Cajal Health Research Institute (IRYCIS), 28034 Madrid, Spain

**Keywords:** flavonoids, aglycons, glycosides, IL-1β, TNF-α, IL-6, IL-8, pro-inflammatory cytokines

## Abstract

Systemic inflammation, circulating immune cell activation, and endothelial cell damage play a critical role in vascular pathogenesis. Flavonoids have shown anti-inflammatory effects. In this study, we investigated the effects of different flavonoids on the production of pro-inflammatory interleukin (IL) 1β, 6, and 8, and tumor necrosis factor α (TNF-α), in peripheral blood cells. Methods: We studied the whole blood from 36 healthy donors. Lipopolysaccharide (LPS)-stimulated (0.5 μg/mL) whole-blood aliquots were incubated in the presence or absence of different concentrations of quercetin, rutin, naringenin, naringin, diosmetin, and diosmin for 6 h. Cultures were centrifuged and the supernatant was collected in order to measure IL-1β, TNF-α, IL-6, and IL-8 production using specific immunoassay techniques. This production was significantly inhibited by quercetin, naringenin, naringin, and diosmetin, but in no case by rutin or diosmin. Flavonoids exert different effects, maybe due to the differences between aglycons and glucosides present in their chemical structures. However, these studies suggest that quercetin, naringenin, naringin, and diosmetin could have a potential therapeutic effect in the inflammatory process of cardiovascular disease.

## 1. Introduction

Cardiovascular diseases are the number one cause of death in the world [1]. Nowadays, the important role of inflammatory events in cardiovascular diseases is widely known [2]. Systemic inflammation, circulating immune cell activation, and endothelial cell damage are critical events, along with arterial wall damage [3,4,5,6]. Furthermore, the relevance of inflammation in the pathogenesis of chronic venous disorder has also been shown [7,8]. Monocytes play a critical role in the inflammatory response [9]. Activated monocytes display relevant immunomodulatory activities, including the secretion of pivotal cytokines, such as pro-inflammatory cytokines interleukin (IL)-6, IL-1β, IL-8, and tumor necrosis factor α (TNF-α). Different mechanisms may be involved in the abnormal activation of monocytes in chronic diseases [10]. There is increasing evidence that gut dysbiosis; increased intestinal permeability, also known as “leaky gut”; and bacterial translocation, are key mechanisms in the induction of the systemic increase of lipopolysaccharide (LPS) and pro-inflammatory monocyte activation in several chronic diseases [11,12,13]. LPS is a potent signal for monocyte stimulation [14]. Monocytes have been demonstrated to be involved in the pathogenesis of several cardiovascular diseases, such as atherosclerosis [15].

Flavonoids are the largest group of naturally occurring polyphenolic compounds, present in almost all parts of flowering plants [16]. Their basic chemical structure is made up of two aromatic rings (rings A and B) connected by an oxygen-containing pyran ring (ring C). Flavonoids are classified into different subgroups, including flavones, isoflavones, flavonols, dihydroflavones, flavanes, chalcones, and anthocyanidins, according to the hydroxylation pattern of rings A and B, the oxidative degree of ring C, and the structure and position of the substitutions [17].

The structural diversity of flavonoids results in a wide range of biological effects; the different substitutions on the carbon atoms determine the biological effects of the flavonoids [18]. It has been reported that flavones show different pharmacological actions, such as antinociceptive [19], anti-inflammatory [20], antioxidant [21], antiulcerogenic [22], and anticarcinogenic [23] actions. The combination of multiple pharmacological properties in a single nucleus is quite interesting [16]. Several mechanisms have been shown to be involved in the anti-inflammatory effects of flavonoids. Flavonoids have the ability to modulate macrophages from pro- to anti-inflammatory phenotypes, potentially contributing to the resolution of pre-established inflammatory processes [24,25]. Furthermore, flavonoids have inhibitory effects in platelet activation, being critical cells for vascular inflammation [26].

The therapeutic activity of flavonoids has been suggested for inflammatory diseases such as cardiovascular diseases, obesity, diabetes, bone health, and asthma [27,28,29,30,31]. Furthermore, the potential use of these molecules in the adjuvant treatment of cancer has also been suggested [25,32]. 

Flavonoids are secondary metabolites in plants, occurring in virtually all plant parts; especially photosynthesizing plant cells [33]. Polyphenols represent the most abundant compounds among secondary metabolites produced by plants. Naringenin, diosmetin, and quercetin are the aglycons of different kinds of flavonoids found in their heteroside form, specifically naringin, diosmin, and rutin. Naringin and its aglycon naringenin are the most important dihydroflavones that have been isolated from citrus fruits [34]. Quercetin and rutin are two flavonols widely distributed among plants and commonly found in daily diets, predominantly in fruits and vegetables [35,36]. On the other hand, diosmin and diosmetin are two flavones found in various dietary sources, such as oregano spice; oregano leaves; citrus fruits; and extracts from specific medicinal herbs of Rosaceae, Asteraceae, Brassicaceae, and Caryophyllaceae [37].

A flavonoid’s biological in vivo activity is very dependent on its bioavailability and this is determined by the chemical structure, mainly the type of sugar moiety. In general, the glycoside levels in plasma are low. Deglycosylation occurs both in the small intestine and in the large intestine, depending on the type of sugar moiety and the aglycon produced by the microbiota. Then, metabolites are absorbed via the large intestine and transported into the circulation [38].

It is possible to hypothesize that flavonoids might inhibit the production of pro-inflammatory cytokines through the activation of leukocytes and focusing on those that are mainly secreted by activated monocytes. The aim of this work was to evaluate the immunomodulatory effect of quercetin, rutin, naringenin, naringin, diosmetin, and diosmin on the production of pro-inflammatory cytokines TNF-α, IL-1β, IL-6, and IL-8 in whole-blood cells stimulated by LPS. 

## 2. Results 

### 2.1. Time Course Cytokine Production Curves 

**Figure 1:**
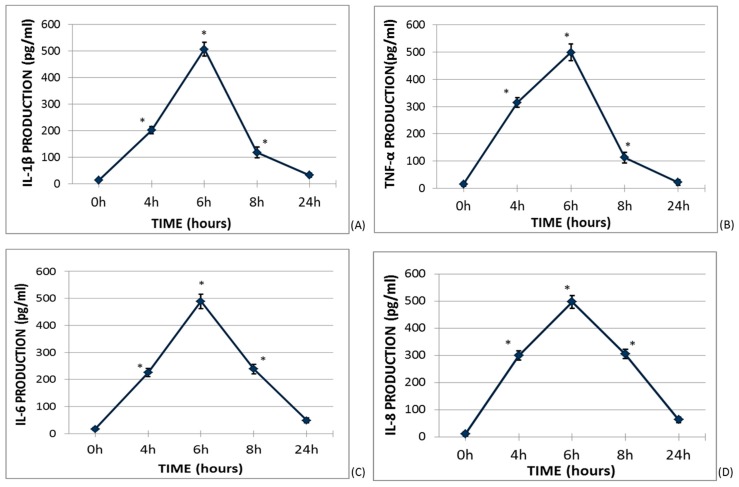
Cytokine production curves in lipopolysaccharide (LPS)-stimulated whole blood. One milliliter of whole blood was incubated in the presence of LPS (0.5 μg/mL) in darkness and continuously shaken at 37 °C. The supernatant concentrations of interleukin (IL)-1β, tumor necrosis factor α (TNF-α), IL-6, and IL-8 were measured by ELISA at 0, 4, 6, 8, and 24 h of culture. The diamonds and vertical segments represent the mean ± SEM of six different donors. * *p* < 0.05 represents a significant difference each time the measurement was compared to baseline production.

Firstly, the kinetic of cytokines produced by lipopolysaccharide (LPS)-stimulated whole blood was investigated (Figure 1). The culture medium concentration of IL-1β, TNF-α, IL-6, and IL-8 at basal conditions and after 4, 6, 8, and 24 h was quantified. The maximum cytokine concentration was found at 6h of culture and this time was chosen as the time condition for subsequent assays.

### 2.2. Study of the Effects of Flavonoids in Cytokine Production in LPS-Stimulated Whole Blood

**Figure 2:**
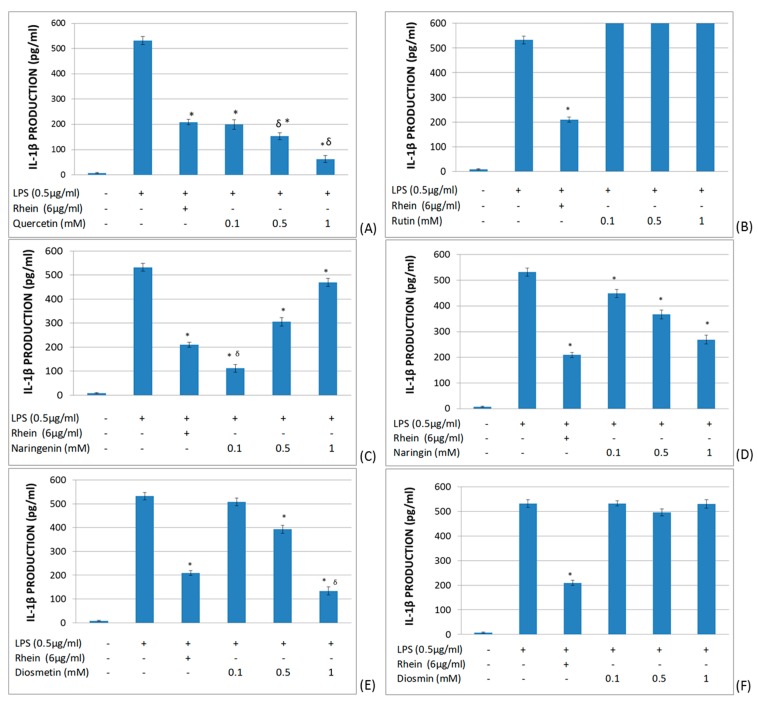
Effects of different flavonoids on the production of IL-1β in LPS-stimulated (0.5 μg/mL) whole blood after 6 h of culture. The different panels show the results of the effects of quercetin (**A**), rutin (**B**), naringenin (**C**), naringin (**D**), diosmetin (**E**), and diosmin (**F**). All data are expressed as the mean (top segment of the rectangles) ± SEM (vertical segment) of thirty independent experiments. * *p* < 0.05: significantly different when compared to the LPS control. δ*p* < 0.05: significantly different when compared to the rhein control.

The effects of quercetin, rutin, naringenin, naringin, diosmetin, and diosmin on IL-1β production in LPS-stimulated whole blood (Figure 2) were investigated. As a control, the inhibitor of IL-1β rhein (diacerhein-derived metabolite, inhibitor of IL-1β production) was used [39]. It was found that quercetin, diosmetin, and naringin significantly reduced IL-1β production in a dose-dependent manner. Naringenin also significantly inhibited IL-1β production, but in an inverse dose-dependent manner. In contrast, rutin and diosmin did not modify IL-1β production in LPS-stimulated whole blood. 

**Figure 3:**
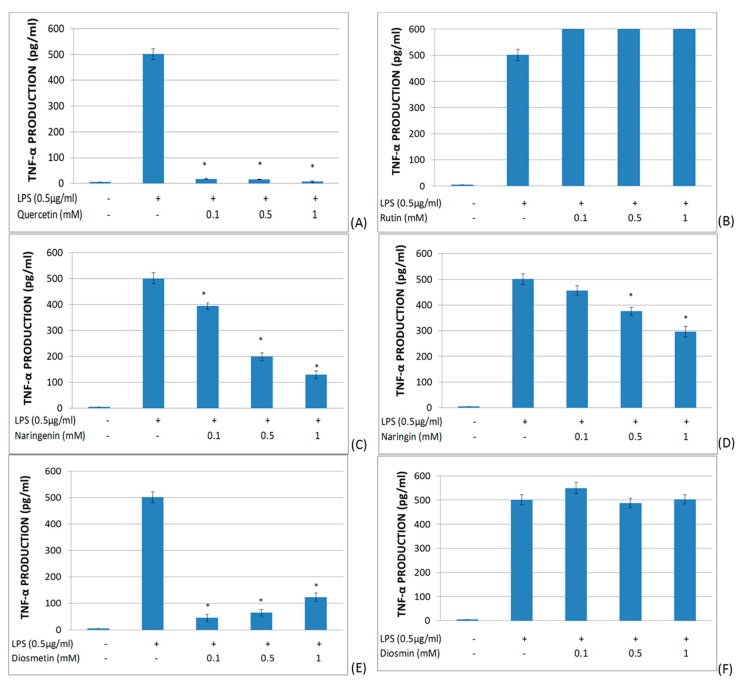
Effects of different flavonoids on the production of TNF-α in LPS-stimulated (0.5 μg/mL) whole blood after 6 h of culture. The different panels show the results of the effects of quercetin (**A**), rutin (**B**), naringenin (**C**), naringin (**D**), diosmetin (**E**), and diosmin (**F**). All data are expressed as the mean (top segment of the rectangles) ± SEM (vertical segment) of thirty independent experiments. * *p* < 0.05: significantly different when compared to the LPS control.

The effects of quercetin, rutin, naringenin, naringin, diosmetin, and diosmin on the TNF-α secretion in LPS-stimulated whole blood (Figure 3) were investigated. Quercetin showed a dramatic inhibitory effect on TNF-α production. Both naringenin and naringin showed a dose-dependent suppressor effect upon TNF-α production. Diosmetin significantly inhibited TNF-α production, but in an inverse dose-dependent manner. On the other hand, rutin and diosmin did not modify TNF-α production in LPS-stimulated whole blood.

**Figure 4:**
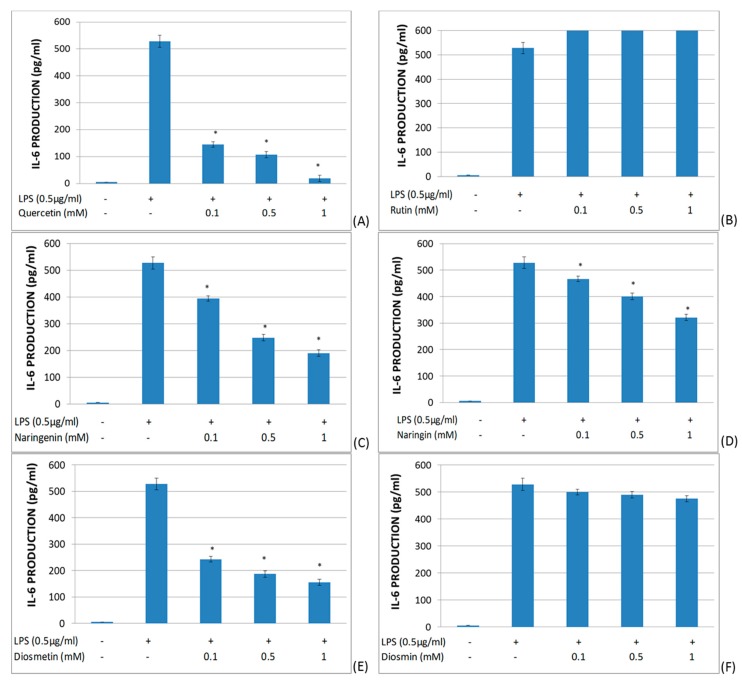
Effects of different flavonoids on the production of IL-6 in LPS-stimulated (0.5 μg/mL) whole blood after 6 h of culture. The different panels show the results of the effects of quercetin (**A**), rutin (**B**), naringenin (**C**), naringin (**D**), diosmetin (**E**), and diosmin (**F**). All data are expressed as the mean (top segment of the rectangles) ± SEM (vertical segment) of thirty independent experiments. * *p* < 0.05: significantly different when compared to the LPS control.

The effects of quercetin, rutin, naringenin, naringin, diosmetin, and diosmin on IL-6 secretion in LPS-stimulated whole blood were studied (Figure 4). It was found that quercetin, naringenin, diosmetin, and naringin significantly reduced IL-6 production in a dose-dependent manner. On the other hand, rutin and diosmin did not significantly modify the IL-6 production in LPS-stimulated whole blood.

**Figure 5:**
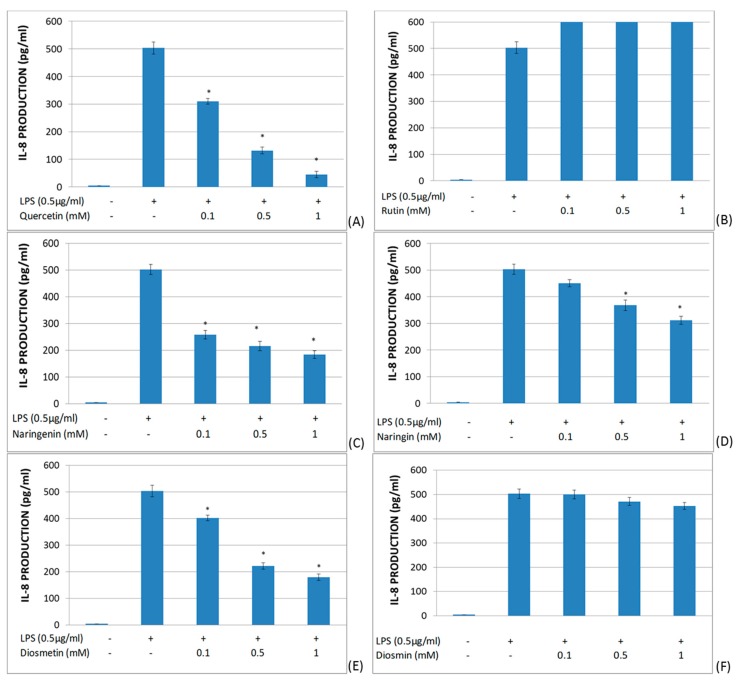
Effects of different flavonoids on the production of IL-8 in LPS-stimulated (0.5 μg/mL) whole blood after 6 h of culture. The different panels show the results of the effects of quercetin (**A**), rutin (**B**), naringenin (**C**), naringin (**D**), diosmetin (**E**), and diosmin (**F**). All data are expressed as the mean (top segment of the rectangles) ± SEM (vertical segment) of thirty independent experiments. * *p* < 0.05: significantly different when compared to the LPS control.

The effect of quercetin, rutin, naringenin, naringin, diosmetin, and diosmin on IL-8 secretion in LPS-stimulated whole blood was studied (Figure 5). In these assays, quercetin, naringenin, diosmetin, and naringin showed a significant decrease in IL-8 production in a dose-dependent manner. Furthermore, rutin and diosmin did not alter IL-8 production in LPS-stimulated whole blood.

## 3. Discussion

In this work, we have demonstrated that certain members of the flavonoid family have an inhibitory effect on the production of IL-1β, TNF-α, IL-6, and IL-8 in LPS-stimulated whole blood. Quercetin, naringenin, naringin, and diosmetin have this immunosuppressor effect. However, rutin and diosmin lack this anti-inflammatory regulatory effect. 

**Figure 6:**
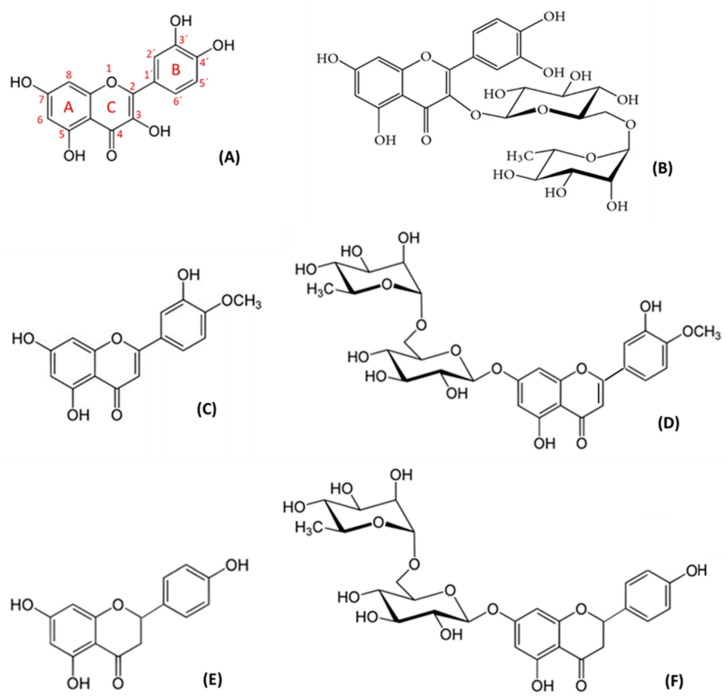
Chemical structure of flavonoids: (**A**) Quercetin, (**B**) rutin, (**C**) diosmetin, (**D**) diosmin, (**E**) naringenin, and (**F**) naringin.

The inflammatory-immune system plays a critical role in the pathogenesis of atherosclerosis [40]. Large thrombogenic necrotic cores, along with lipids, intraplaque hemorrhage, a thinner fibrous cap, and inflammatory cell infiltration, pathologically characterize unstable plaque rupture, and these pathological features eventually result in platelet aggregation and thrombus formation [41]. Control of the molecular and cellular mechanisms involved in these processes is an area of intense research. Some flavonoids have shown the ability to inhibit platelet activation and aggregation [26]. In this study, we have further investigated the anti-inflammatory and immunoregulatory effects of flavonoids, focusing on the production of pro-inflammatory cytokines in LPS-stimulated whole blood. LPS is the principal component of the outer membrane of gram-negative bacteria, and one of the most potent inflammation inducers of pro-inflammatory cytokines [42]. LPS is a relevant monocyte activation signal with a marked effect on the production of inflammatory cytokines, including IL-1β, TNF-α, IL-6, and IL-8 [43]. The effect of flavonoids varies, depending on their chemical structures and functional groups [18]. The presence of C3–hydroxyl (OH) may result in an anti-inflammatory effect. Indeed, quercetin, which has a C3–OH group (the characteristic of flavonols), enhanced LPS-stimulated cytokine inhibition. The other aglycons studied, like naringenin and diosmetin, demonstrated a relevant cytokine suppressor effect. Diosmetin is a flavone which contains a carbon–carbon double bond (C2=C3) in ring C, and a C5–OH and C7–OH group in ring A (Figure 6). The dihydroflavone naringenin differs from diosmetin by the presence of a carbon–carbon single bond (C2–C3), and by the different substitutions on the carbon atoms of ring B (Figure 6). The glycoside naringin is a dihydroflavone which has glycosylation on C7–OH (Figure 6) and exerted a cytokine-suppressor effect, but one that was not as relevant as the aglycons. However, rutin and diosmin did not show any inhibitory capacity in the release of pro-inflammatory cytokines. Rutin, a flavonol with glycosylation of C3–OH (Figure 6), did not have any effect on the LPS-induced release of cytokines, similar to diosmin, which belongs to the flavones group, with the glycosylation of C7–OH (Figure 6).

It is well-known that disaccharide structures of glycosides block the anti-inflammatory activity. There are works on the metabolism of flavonoids and the major effect of their metabolites in the organism [44,45]. Aglycons may enter epithelial cells by passive diffusion because of their increased lipophilicity. Therefore, generally, flavonoid glycosides are cleaved either in the intestinal lumen or in the epithelium before absorption [46]. In vitro, according to our results, aglycons exert higher activity versus heterosides. In this sense, quercetin has five hydroxyl groups (Figure 6) capable of interacting with the receptor binding site, and one of them is in the most active position: the C3–OH on ring C [47]. Due to these structural characteristics, quercetin has shown an intense and wide inhibitory effect on whole-blood production of TNF-α at all assayed concentrations. These inhibitory results of quercetin are also remarkable in the studied interleukin release. Likewise, diosmetin has three hydroxyl groups, which could justify its inhibitory effect, although this effect is lower than that of quercetin, since none of the OH is located at the 3 position. The inhibitory effect of diosmetin is exerted in a dose-dependent manner for IL-1β, IL-6, and IL-8 production; however, the release of TNF-α is inhibited in an inverse dose-dependent manner. Naringenin also contains three hydroxyl groups in its structure, and no C3–OH on ring C. This product inhibits, in a dose-dependent manner, IL-6, IL-8, and TNF-α, and in an inverse dose-dependent manner, IL-1β. Rutin and diosmin did not exert any activity in our in vitro assays, maybe due to their disaccharide structures, although naringin, despite containing disaccharides in its structure, showed some activity. 

On the other hand, the basic structure of flavonoids and which type of sugar moiety is attached strongly affect their bioavailability. Bioavailability is a crucial factor determining their biological activity in vivo. Dietary flavonoids are mostly present in their glycoside forms. However, this is not the case in the plasma, where glycosides are scarce. As we discussed previously, deglycosylation occurs both in the small intestine and in the large intestine, depending on the type of sugar moiety. In the small intestine, two enzymes have been reported to act, such as β-glucosidases, against flavonoid monoglucosides. 

In the case of non-monoglucosidic glycosides of flavonols, such as rutin, intestinal β-glucosidases cannot hydrolyze the sugar moiety. Therefore, the intestinal microbiota acts to yield absorbable aglycon in the cecum and in the large intestine. The aglycon produced by the microbiota is absorbed via the large intestine and transported into the circulation [38]. Regarding flavones, such as diosmin, the oral absorption is poor, reaching plasma concentrations that are typically < 1 μg/mL in humans. However, formulations of micronized diosmin have improved the bioavailability and they are widely used in the treatment of chronic venous disease, hemorrhoidal disease, and other indications [48]. Numerous studies have confirmed the superior efficacy of micronized diosmin (therapeutic dose = 1000 mg) compared to non-micronized diosmin (1000 mg), through the diosmetin plasma concentration as the main metabolic by-product of diosmin [49]. There were no significant adverse events in the study groups [48].

Regarding dihydroflavones, naringin in humans is metabolized in aglycon naringenin by naringinase found in the liver. Its solubility is limited in water and has a low oral bioavailability of around 5% [50]. Dihydroflavones are absorbed into enterocytes after oral intake; they are rapidly metabolized, in particular, into conjugates, sulfates, and glucuronides, which are the major circulating forms in plasma. A large fraction reaches the colon, where it is efficiently metabolized into small absorbable phenolics. The form (aglycon vs. glycoside) and species (e.g., human vs. rat) have important impacts [51].

These results support the anti-inflammatory and immunomodulatory effects of flavonoids, in addition to their role in platelet activation [26]. Nowadays, it is not possible to reach high concentrations in plasma due to the lack of flavonoid solubility, but these assays hint at clarifying the possible mechanism of action of these compounds. Further studies are required to define the different cellular targets of flavonoids in pro-inflammatory cytokines. It is possible to suggest in our experimental system that monocytes, as LPS responders, are potential targets of the flavonoid immunomodulatory activity. Additional studies may establish the potential therapeutic relevance of these flavonoid effects in vascular diseases. 

## 4. Materials and Methods

### 4.1. Study Cohort, and Inclusion and Exclusion Criteria

Forty healthy non-smokers (age 21.8 ± 1.01 years (mean ± SEM); 11 men, 25 women) provided blood for the present study. Possible donors were excluded if they showed evidence of heart, kidney, lung, or autoimmune disease; had a history of tumors, any chronic or acute infection, diabetes mellitus, hypercholesterolemia, endocrine diseases, immunodeficiency, or thrombocytopathy; were undergoing immunosuppressant, immunomodulatory, cytostatic, or nonsteroidal anti-inflammatory drug (NSAID) treatment; or took any other medication within the three months prior to the study that might modify the cytokine response.

Written informed consent was obtained from each donor. The study was conducted according to the ethical guidelines of the 1975 Declaration of Helsinki, with the approval of the Biomedical Ethics Committee of the University of Alcalá de Henares. 

### 4.2. Peripheral Blood Extraction

Peripheral blood was collected by antecubital puncture in sodium citrate-containing (3.8% wt/vol) Vacutainer^®^ tubes (Dismadel, Spain), discarding the first 2 mL. All extractions were performed at the Dept. of Haematology of the *Principe de Asturias Hospital*, Alcala de Henares (Spain). Sodium citrate was selected as the anticoagulant instead of heparin, ethylenediaminetetraacetic acid (EDTA), or d-phenylalanyl-l-prolyl-l-arginine chloromethyl ketone (PPACK), given its lesser impact on complement activation pathways [52].

### 4.3. Selected Drugs

Quercetin (Sigma-Aldrich Chemical, Spain), rutin (Sigma-Aldrich Chemical, Spain), diosmetin (Zoster Ferrer, Spain), diosmin (Zoster Ferrer, Spain), naringenin (Zoster Ferrer, Spain), and naringin (Zoster Ferrer, Spain) (Figure 6) were dissolved in dimethyl sulphoxide (DMSO) (Dismadel, Spain) to a final plasmatic concentration of 0.5 mM. This concentration is close to the clinical dose used in the flavone diosmin (Daflon^®^) [53]. The difficulty in dissolving the compounds meant that no concentrations higher than 1mM could be tested. Then, further dilutions of 0.1, 0.5, and 1 mM were produced for use in later assays. The smallest volume of DMSO (2 µL), which allowed a perfect dissolution of compounds, was added to blood to avoid modifying or changing the cell structure.

### 4.4. Assay of IL-1β, TNF-α, Il-6, and IL-8 Production in LPS-Stimulated Whole Blood

Whole-blood aliquots of 1mL, extracted from donors, were incubated with flavonoids at 37 °C for 30 min in darkness and with continuous shaking. Afterwards, 0.5 μg/mL of lipopolysaccharide was added, to boost the production of different cytokines by monocytes [54], and incubated at 37 °C for 6 h in darkness and with continuous shaking.

Next, samples were introduced in dry ice and were gradually centrifuged (Centrifugal machine JOUAN B3.11 model) for 10 min at 4000 rpm (revolutions per minute), and then, the supernatant was collected. Every control and product was assayed in quadruplicate.

The levels of different cytokines were measured by using a specific enzyme immunoassay kit for each cytokine (IL-1β Biotrak ELISA kit, Amersham Biosciences, Little Chalfont, UK; TNF-α Biotrak ELISA kit, Amersham Biosciences, Little Chalfont, UK; Il-6 Biotrak ELISA kit, Amersham Biosciences, Little Chalfont, UK; IL_8 Biotrak ELISA kit, Amersham Biosciences, Little Chalfont, UK), in accordance with the manufacturer’s instructions. Absorbance was measured with a spectrophotometer (ELx800 Absorbance Microplate Reader of Biotek, Wisconsin, USA) at 450 nm. The standard curve for cytokines covered the range from 7.8 to 500 pg/mL. The intra- and inter-assay coefficients of variation (CVs) were 7.6% and 10.3%, respectively. The assay sensitivity was 1.1 pg/mL. 

Dimethyl sulfoxide (DMSO) and other reagents, unless specifically stated elsewhere, were purchased from Sigma-Aldrich (St. Louis, MO, USA). The final volume of DMSO in the reaction mixture was 0.5%.

### 4.5. Statistical Analysis

All results are expressed as the mean ± SEM (standard error mean) of values obtained in each experiment. Since most variables did not fulfil the normality hypothesis, a Wilcoxon test was used to analyse the variance of paired groups. The significance level was set at *p* < 0.05. Statistical analysis was performed using SPSS-19 software (SPSS-IBM, Armonk, NY, USA).

## References

[1] Kaptoge S., Pennells L., De Bacquer D., Cooney M.T., Kavousi M., Stevens G., Riley L.M., Savin S., Khan T., Altay S. (2019). World Health Organization cardiovascular disease risk charts: Revised models to estimate risk in 21 global regions. Lancet Glob. Health.

[2] Becker R.C., Owens A.P., Sadayappan S. (2020). Tissue-level inflammation and ventricular remodeling in hypertrophic cardiomyopathy. J. Thromb Thrombolysis.

[3] Melnikov I.S., Kozlov S.G., Saburova O.S., Avtaeva N.Y., Prokofieva L.V., Gabbasov Z.A. (2019). Current position on the role of monomeric C-reactive protein in vascular pathology and atherothrombosis. CPD.

[4] Chiu Y.-J., Hsieh Y.-H., Lin T.-H., Lee G.-C., Hsieh-Li H.M., Sun Y.-C., Chen C.-M., Chang K.-H., Lee-Chen G.-J. (2019). Novel compound VB-037 inhibits Aβ aggregation and promotes neurite outgrowth through enhancement of HSP27 and reduction of P38 and JNK-mediated inflammation in cell models for Alzheimer’s disease. Neurochem. Int..

[5] Vogel S., Thein S.L. (2018). Platelets at the crossroads of thrombosis, inflammation and haemolysis. Br. J. Haematol.

[6] Mkhize N.V.P., Qulu L., Mabandla M.V. (2017). The Effect of Quercetin on Pro- and Anti-Inflammatory Cytokines in a Prenatally Stressed Rat Model of Febrile Seizures. J. Exp. Neurosci..

[7] Ortega M.A., Asúnsolo Á., Romero B., Álvarez-Rocha M.J., Sainz F., Leal J., Álvarez-Mon M., Buján J., García-Honduvilla N. (2018). Unravelling the Role of MAPKs (ERK1/2) in Venous Reflux in Patients with Chronic Venous Disorder. Cells Tissues Organs.

[8] Ortega M.A., Asúnsolo Á., Leal J., Romero B., Alvarez-Rocha M.J., Sainz F., Álvarez-Mon M., Buján J., García-Honduvilla N. (2018). Implication of the PI3K/Akt/mTOR Pathway in the Process of Incompetent Valves in Patients with Chronic Venous Insufficiency and the Relationship with Aging. Oxidative Med. Cell. Longev..

[9] Colmorten K.B., Nexoe A.B., Sorensen G.L. (2019). The Dual Role of Surfactant Protein-D in Vascular Inflammation and Development of Cardiovascular Disease. Front. Immunol..

[10] Kuznetsova T., Prange K.H.M., Glass C.K., de Winther M.P.J. (2019). Transcriptional and epigenetic regulation of macrophages in atherosclerosis. Nat. Rev. Cardiol.

[11] Alvarez-Mon M.A., Gómez A.M., Orozco A., Lahera G., Sosa M.D., Diaz D., Auba E., Albillos A., Monserrat J., Alvarez-Mon M. (2019). Abnormal Distribution and Function of Circulating Monocytes and Enhanced Bacterial Translocation in Major Depressive Disorder. Front. Psychiatry.

[12] Jayashree B., Bibin Y.S., Prabhu D., Shanthirani C.S., Gokulakrishnan K., Lakshmi B.S., Mohan V., Balasubramanyam M. (2014). Increased circulatory levels of lipopolysaccharide (LPS) and zonulin signify novel biomarkers of proinflammation in patients with type 2 diabetes. Mol. Cell. Biochem..

[13] Sieve I., Ricke-Hoch M., Kasten M., Battmer K., Stapel B., Falk C.S., Leisegang M.S., Haverich A., Scherr M., Hilfiker-Kleiner D. (2018). A positive feedback loop between IL-1β, LPS and NEU1 may promote atherosclerosis by enhancing a pro-inflammatory state in monocytes and macrophages. Vasc. Pharmacol..

[14] Murray P.J. (2018). Immune regulation by monocytes. Semin. Immunol..

[15] Swirski F.K., Nahrendorf M. (2013). Leukocyte Behavior in Atherosclerosis, Myocardial Infarction, and Heart Failure. Science.

[16] Umamaheswari S. (2015). Anti-Inflammatory Effect of Selected Dihydroxyflavones. JCDR.

[17] González R., Ballester I., López-Posadas R., Suárez M.D., Zarzuelo A., Martínez-Augustin O., Medina F.S.D. (2011). Effects of Flavonoids and other Polyphenols on Inflammation. Crit. Rev. Food Sci. Nutr..

[18] Dymarska M., Janeczko T., Kostrzewa-Susłow E. (2018). Glycosylation of Methoxylated Flavonoids in the Cultures of Isaria fumosorosea KCH J2. Molecules.

[19] Justino A.B., Costa M.S., Saraiva A.L., Silva P.H., Vieira T.N., Dias P., Linhares C.R.B., Dechichi P., de Melo Rodrigues Avila V., Espindola F.S. (2019). Protective effects of a polyphenol-enriched fraction of the fruit peel of Annona crassiflora Mart. on acute and persistent inflammatory pain. Inflammopharmacology.

[20] Domingos O.D.S., Alcântara B.G.V., Santos M.F.C., Maiolini T.C.S., Dias D.F., Baldim J.L., Lago J.H.G., Soares M.G., Chagas-Paula D.A. (2019). Anti-Inflammatory Derivatives with Dual Mechanism of Action from the Metabolomic Screening of Poincianella pluviosa. Molecules.

[21] Saha S., Panieri E., Suzen S., Saso L. (2020). The Interaction of Flavonols with Membrane Components: Potential Effect on Antioxidant Activity. J. Membr. Biol.

[22] Boligon A.A., de Freitas R.B., de Brum T.F., Waczuk E.P., Klimaczewski C.V., de Ávila D.S., Athayde M.L., de Freitas Bauermann L. (2014). Antiulcerogenic activity of Scutia buxifolia on gastric ulcers induced by ethanol in rats. Acta Pharm. Sin. B.

[23] Li X., Sdiri M., Peng J., Xie Y., Yang B.B. (2020). Identification and characterization of chemical components in the bioactive fractions of *Cynomorium coccineum* that possess anticancer activity. Int. J. Biol. Sci..

[24] Mendes L.F., Gaspar V.M., Conde T.A., Mano J.F., Duarte I.F. (2019). Flavonoid-mediated immunomodulation of human macrophages involves key metabolites and metabolic pathways. Sci Rep..

[25] Magne Nde C.B., Zingue S., Winter E., Creczynski-Pasa T.B., Michel T., Fernandez X., Njamen D., Clyne C. (2015). Flavonoids, Breast Cancer Chemopreventive and/or Chemotherapeutic Agents. Curr. Med. Chem..

[26] Zaragozá C., Monserrat J., Mantecón C., Villaescusa L., Zaragozá F., Álvarez-Mon M. (2016). Antiplatelet activity of flavonoid and coumarin drugs. Vasc. Pharmacol..

[27] Khalilpourfarshbafi M., Gholami K., Murugan D.D., Abdul Sattar M.Z., Abdullah N.A. (2019). Differential effects of dietary flavonoids on adipogenesis. Eur J. Nutr.

[28] Meng H., Shao D., Li H., Huang X., Yang G., Xu B., Niu H. (2018). Resveratrol improves neurological outcome and neuroinflammation following spinal cord injury through enhancing autophagy involving the AMPK/mTOR pathway. Mol Med. Rep..

[29] Tanaka T., Takahashi R. (2013). Flavonoids and Asthma. Nutrients.

[30] Che C.-T., Wong M., Lam C. (2016). Natural Products from Chinese Medicines with Potential Benefits to Bone Health. Molecules.

[31] Gómez-Guzmán M., Rodríguez-Nogales A., Algieri F., Gálvez J. (2018). Potential Role of Seaweed Polyphenols in Cardiovascular-Associated Disorders. Mar. Drugs.

[32] Gonçalves C., de Freitas M., Ferreira A. (2017). Flavonoids, Thyroid Iodide Uptake and Thyroid Cancer—A Review. IJMS.

[33] Kumar S., Pandey A.K. (2013). Chemistry and Biological Activities of Flavonoids: An Overview. Sci. World J..

[34] Tripoli E., Guardia M.L., Giammanco S., Majo D.D., Giammanco M. (2007). Citrus flavonoids: Molecular structure, biological activity and nutritional properties: A review. Food Chem..

[35] Khan H., Ullah H., Aschner M., Cheang W.S., Akkol E.K. (2019). Neuroprotective Effects of Quercetin in Alzheimer’s Disease. Biomolecules.

[36] Heim K.E., Tagliaferro A.R., Bobilya D.J. (2002). Flavonoid antioxidants: Chemistry, metabolism and structure-activity relationships. J. Nutr. Biochem..

[37] Doostdar H., Burke M.D., Mayer R.T. (2000). Bioflavonoids: Selective substrates and inhibitors for cytochrome P450 CYP1A and CYP1B1. Toxicology.

[38] Murota K., Nakamura Y., Uehara M. (2018). Flavonoid metabolism: The interaction of metabolites and gut microbiota. Biosci. Biotechnol. Biochem..

[39] De Isla N.G., Yang J.W., Huselstein C., Muller S., Stoltz J.F. (2006). IL-1beta synthesis by chondrocyte analyzed by 3D microscopy and flow cytometry: Effect of Rhein. Biorheology.

[40] Pescetelli I., Zimarino M., Ghirarduzzi A., De Caterina R. (2015). Localizing factors in atherosclerosis. J. Cardiovasc. Med..

[41] Sun B., Zhao H., Li X., Yao H., Liu X., Lu Q., Wan J., Xu J. (2017). Angiotensin II-accelerated vulnerability of carotid plaque in a cholesterol-fed rabbit model-assessed with magnetic resonance imaging comparing to histopathology. Saudi J. Biol. Sci..

[42] Dholakiya S.L., Benzeroual K.E. (2011). Protective effect of diosmin on LPS-induced apoptosis in PC12 cells and inhibition of TNF-α expression. Toxicology in Vitro.

[43] Lee S.-B., Lee W.S., Shin J.-S., Jang D.S., Lee K.T. (2017). Xanthotoxin suppresses LPS-induced expression of iNOS, COX-2, TNF-α, and IL-6 via AP-1, NF-κB, and JAK-STAT inactivation in RAW 264.7 macrophages. Int. Immunopharmacol..

[44] Serreli G., Deiana M. (2019). In vivo formed metabolites of polyphenols and their biological efficacy. Food Funct..

[45] Olivares-Vicente M., Barrajon-Catalan E., Herranz-Lopez M., Segura-Carretero A., Joven J., Encinar J.A., Micol V. (2018). Plant-Derived Polyphenols in Human Health: Biological Activity, Metabolites and Putative Molecular Targets. Curr. Drug Metab..

[46] Cassidy A., Minihane A.-M. (2017). The role of metabolism (and the microbiome) in defining the clinical efficacy of dietary flavonoids. Am. J. Clin. Nutr..

[47] Heřmánková E., Zatloukalová M., Biler M., Sokolová R., Bancířová M., Tzakos A.G., Křen V., Kuzma M., Trouillas P., Vacek J. (2019). Redox properties of individual quercetin moieties. Free Radic. Biol. Med..

[48] Martel C., Cointe S., Maurice P., Matar S., Ghitescu M., Théroux P., Bonnefoy A. (2011). Requirements for membrane attack complex formation and anaphylatoxins binding to collagen-activated platelets. PLoS ONE.

[49] Staniewska A. (2016). Safety of use of micronized diosmin at daily doses up to 2000 mg per day. Pol. Merkur. Lek..

[50] Russo R., Chandradhara D., De Tommasi N. (2018). Comparative Bioavailability of Two Diosmin Formulations after Oral Administration to Healthy Volunteers. Molecules.

[51] Ratnam D.V., Ankola D.D., Bhardwaj V., Sahana D.K., Kumar M.N.V.R. (2006). Role of antioxidants in prophylaxis and therapy: A pharmaceutical perspective. J. Control. Release.

[52] Najmanová I., Vopršalová M., Saso L., Mladěnka P. (2019). The pharmacokinetics of flavones. Crit Rev. Food Sci Nutr.

[53] Bogucka-Kocka A., Woźniak M., Feldo M., Kockic J., Szewczyk K. (2013). Diosmin--isolation techniques, determination in plant material and pharmaceutical formulations, and clinical use. Nat. Prod. Commun.

[54] Lima T.S., Gov L., Lodoen M.B. (2018). Evasion of Human Neutrophil-Mediated Host Defense during Toxoplasma gondii Infection. mBio.

